# Analysis of Deformation Behaviour and Residual Stress in Rotary Swaged Cu/Al Clad Composite Wires

**DOI:** 10.3390/ma12213462

**Published:** 2019-10-23

**Authors:** Lenka Kunčická, Adéla Macháčková, Ludmila Krátká, Radim Kocich

**Affiliations:** 1 Institute of Physics of Materials, Academy of Sciences of the Czech Republic, Žižkova 22, 61600 Brno, Czech Republic; kuncicka@ipm.cz; 2 VŠB–Technical University of Ostrava, 17. listopadu 15, 70833 Ostrava, Czech Republic; adela.machackova@vsb.cz (A.M.); ludmila.kratka@vsb.cz (L.K.)

**Keywords:** clad composite, rotary swaging, finite element analysis, substructure, residual stress

## Abstract

Both copper and aluminum are widely applicable throughout a variety of industrial and commercial branches, however, joining them in a composite provides the possibility of combining all their advantageous properties in one material. This study investigates uniquely sequenced copper–aluminum clad composite wires, fabricated via rotary swaging technology. The composites were processed at 20 °C and 250 °C to a diameter of 5 mm. Structural observations and the determination of residual stress within both elements of the swaged wires were performed via electron microscopy; the experimental results were correlated with numerical predictions. As shown in the results, both the applied swaging force and temperature affected the plastic flow, which had a direct influence on residual stress and texture development; the Al_sheath_ elements exhibited ideal rolling textures, whereas the Cu_wires_ elements featured ideal shear texture orientation. The grains within both the Al_sheath_ elements of the 5 mm composite wire were refined down to sub-micron size. Structural restoration also had a positive influence on residual stress.

## 1. Introduction

Layered composites, also referred to as clad composites or hybrid materials, are innovative materials consisting of two or more metal bonds at mutual interfaces. Given their ability to simultaneously achieve advantageous mechanical and physical (e.g., thermal and electric.) properties, which is challenging for single-phase materials, an ever-increasing number of commercial and industrial branches are gaining an interest in composites. They are especially favoured for applications in which contradictory material properties are required [1,2]. The application of clad composites makes it possible to combine different properties (e.g., corrosion and wear resistance, thermal and electric resistivity, magnetic properties, and acoustic dumping and energy absorption.) on the inner and outer regions of the individual final product. Clad composites are typically used in the aerospace [3], automotive [4], and marine industries [5], as well as in thermal engineering [6], medicine [7], and electrotechnics—multi-layered materials in the form of layered sheets and clad wires or rods are promising for energy transfer [8].

Composites are typically bi-metallic materials, however, multi-metallic materials and systems combining metals with other compounds have also been studied. A wide spectrum of multi-component materials have been designed and researched [9,10]. The primary research aim has been to optimize composite production, with regard to the achievement of advantageous properties. Nevertheless, economy of production should also be considered, such as reducing the weight of vehicles in the automotive industry, via application of Mg-based alloys. Despite being more promising than, e.g., Al-based alloys, Mg-based alloys have several major drawbacks, such as their relatively low mechanical properties, low formability, and low corrosion resistance, demonstrated by Mg’s tendency to form non-protective surface oxide layers. Based on the results achieved by the coating/cladding of the surfaces of Mg-based alloys with more protective materials, such as aluminum or stainless steel, the production of various clad/layered composites can be considered an effective means of improving the behaviour of materials in, but not limited to, corrosive environments [11,12]. Another example is the effort to reduce the weight and enhance the properties of copper components in electrotechnics by adding aluminum. Recently, Jin et al. [13] produced Al/Cu bimetal tubes via the tube spinning process, in order to perform thorough research on optimized forming parameters, while Kim and Hong [14] produced a rolled Cu/Al/Cu composite sheet to improve the mechanical properties of the individual rolled Cu and Alt—they reported that the fracture strain increased more than 25% compared to the individual metals.

Besides selection of individual materials, selection of suitable production technologies and their optimization has recently been a primary research trend. Among the widely used manufacturing technologies are processes based on welding, e.g., explosion welding [15,16,17], or diffusion welding [18]. However, such processes have certain disadvantages, notably the development of a heat-affected zone, featuring significantly different properties and a different structure, which can negatively influence the performance of the final composite. For example, Yu et.al [19] successfully used underwater explosive cladding to fabricate a Cu/Al cladded tube, however they also found locally developed CuAl_2_ intermetallics. These drawbacks limit the possible usage of the composites for thin and fine components. Nevertheless, alternative means of composite production, via conventional as well as nonconventional methods of severe plastic deformation (SPD), have been developed [20,21].

Regardless the selected forming process, manufacturing technology is always selected with the aim of ensuring the highest possible quality of mutual bonding between the individual components. When formed under cold conditions, clad composites are prone to local debonding at the interfaces. On the other hand, when formed under hot conditions, the elevated temperature can impart the formation and development of intermetallic layers and affect the quality (strength) of the mutual bonding of individual layers [22]. For these reasons, the ideal processing temperature, or optimized heat treatment, is typically searched for.

Drawing has been one of the methods used for fabrication of composites [23], however, it has several drawbacks, which make this method unsuitable for clad composite production. More suitable technology for composite production are the widely used direct and indirect extrusions; the study by Lee at al. [24] documents the fabrication of a Ti/Cu composite via hot indirect extrusion, performed in a vacuum or an inert atmosphere, in order to prevent the oxidation of mutual interfaces. Other popular conventional technologies for layered composite production are rolling and rolling-based methods [25,26,27,28,29]. Although rolling is typically performed in hot conditions, combinations of cold rolling with subsequent heat treatment have also been researched. 

The main advantage of non-conventional SPD methods is their positive effect on the structures of the processed materials—methods like high pressure torsion (HPT) [30,31], Equal channel angular pressing (ECAP) and its modifications [32,33,34,35,36], or friction stir-based methods [37,38], impart formation of ultra-fine grains by imposing severe shear strain, without the need to be processed under hot temperatures. Rotary swaging (RS) and similar intensive plastic deformation methods, such as the accumulative swaging and bundling (ASB) used by Eschke et al. [39] to fabricate a Ti/Al composite, are also promising for the fabrication of clad composites. A primary advantage of RS is the relatively easy production of axi-symmetrical composites of unlimited lengths, featuring fine grains and enhanced properties [40]. Nevertheless, designing an optimum combination of component metals and their sequencing, in order to minimize inhomogeneities in their final properties, is challenging [41]. Also, residual stress can develop during deformation processing, as a result of various influencing factors, such as non-uniform heating/cooling, which typically occurs under cold conditions, or is caused by the inhomogeneous distribution of the imposed strain [42]. 

For these reasons, this study focused on a numerical and experimental evaluation of the presented Cu/Al clad composite, evaluating mechanical behaviour, stress–strain conditions, and texture development.

## 2. Materials and Methods

The initial materials used for the fabrication of the clad composite were commercially pure (CP) Cu (Cu with 0.002% O, 0.015% P, and 0.002% Zn), and electro-conductive CP Al (Al with 0.20% Si, 0.25% Fe, and 0.05% Cu). The initial outer diameter of the Al billet was 30 mm, and the diameters of the inserted Cu wires were 3 mm each. The Al/Cu volume ratio within the clad composite was 81:19.

**Figure 1:**
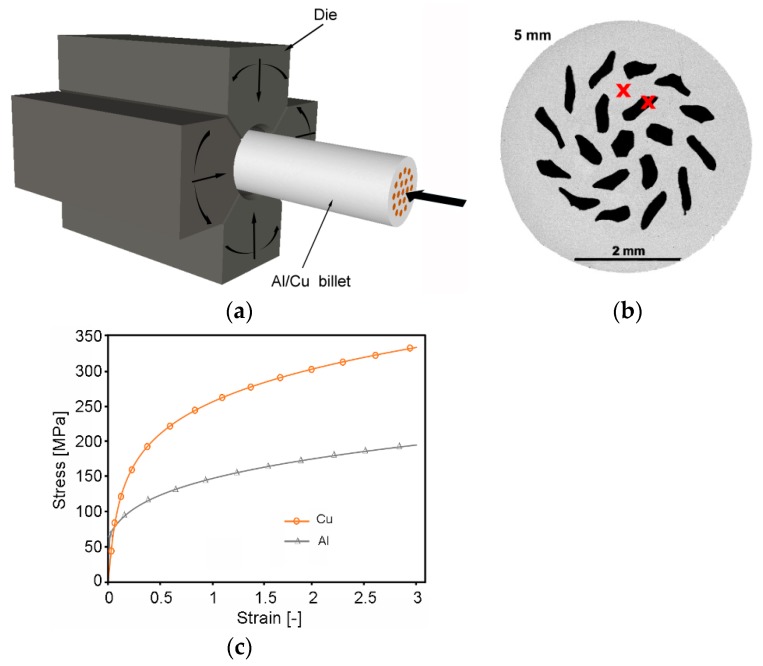
Schematic depiction of rotary swaging setup of Al/Cu composite (**a**); cross-sectional cut of 5 mm 20*C* swaged composite, with locations for structure analyses at approximately mid-radius for both components, marked with “x”; (**b**) stress–strain curves for individual metals used for numerical prediction (**c**).

The 5 mm Al/Cu clad composite wires were manufactured via rotary swaging (RS). The basis of the fabrication technology is schematically depicted in Figure 1a, in which the rotary swaging dies and workpiece can be seen. RS was carried out at a room temperature of approximately 20 °C (composite *20C*), and, at an elevated temperature of 250 °C (composite *250C*), heating was performed by induction, right before the input of the composite into the swaging head. The composites were swaged down to 5 mm in multiple swaging passes. The individual and total reduction ratios depicted in Table 1 were calculated using the *φ* = *ln* (*S*_0_/*S_n_*) relation, where *S*_0_ and *S_n_* were the initial and final composite cross-sections, respectively.

The analyses of the swaged samples were primarily focused on the influence of processing temperature on residual stress and texture development. Thorough observations were carried out via scanning electron microscopy (SEM) and electron backscattered diffraction (EBSD). On the cross-sectional cut of the 5 mm *20C* swaged composite, the locations in which subsequent structure analyses were taken are marked with “x”, as depicted in Figure 1b. The transversal cut samples were mechanically ground on SiC papers and subsequently electrolytically polished. The EBSD analyses were performed with a scan step of 0.25 µm, using a Tescan Lyra 3 equipment (TESCAN Brno s.r.o, Brno, Czech Republic) with a NordlysNano EBSD detector (Oxford Instruments, Abingdon-on-Thames, UK). The scans were evaluated using the ATEX [43] software (Win10 version). Residual stress was evaluated via analyses of internal grain misorientation on a scale of 0° to 15°. Texture was evaluated with a maximum 15° deviation from ideal orientation.

The experiment was supplemented with a numerical prediction focused on the characterization of deformation behaviour and stress-state, and the determination of swaging forces during the first swaging pass. The numerical analysis was performed with the use of FORGE commercial software (NxT version). As seen in Figure 1a, the initial assembly used for the numerical prediction of cold swaging of the composite consisted of four swaging dies, the workpiece, and a clamp bar to feed the workpiece into the swaging head. The workpiece was pushed forward to the head after each stroke —each time the dies were in the open position. All the dies performed a simultaneous oscillation in the radial direction, and a rotation around the swaging axis. The entire assembly was meshed with finite tetrahedron elements; the Al_sheath_ (diameter of 30 mm and length of 100 mm) mesh consisted of 284 125 nodes, and each of the Cu_wires_ was meshed with 12,760 nodes. Given the high number of Al_sheath_/Cu_wire_ interfaces, anisotropic meshing was applied. 

Swaging was carried out at room temperature. The initial temperature of the workpiece and dies was set at 20 °C. Friction between the dies and workpiece was determined by the Coulomb law, with a friction coefficient of μ = 0.1. Material behaviour was defined based on the stress–strain curves acquired via tensile testing of the investigated materials (Figure 1c). The tensile test data were input to the software database and, subsequently, the constitutive model was created. Material behaviour was finally characterized by an elastic–viscoplastic model, determined via the simplified Haensel–Spittel equation defined as Equation (1):

(1) $$\sigma =A\exp(m_{1}T)\varepsilon^{m_{2}}\exp\left(\frac{m_{4}}{\varepsilon }\right){\dot{\varepsilon }}^{m_{3}}$$

where ε is the strain, $\dot{\varepsilon }$ is the strain rate, T is the temperature and A, m_1_, m_2_, m_3_, and m_4_ are regression coefficients. The supplementary thermo-physical parameters ensuing from the model are specified in Table 2.

Among the predicted parameters were the radial swaging forces, which were measured dynamically during the experimental RS, using the KOMAFU S600 system, for dynamic detection of swaging forces with an integrated dynamic measurement of temperature [44,45]. Determination of swaging forces not only indirectly points to the occurrence of strengthening/softening processes within the swaged piece, but also provides the possibility of verifying the performed simulation, which is useful in preventing possible die breakage and improves the safety level of the whole production process.

## 3. Results

### 3.1. Deformation Behaviour

**Figure 2:**
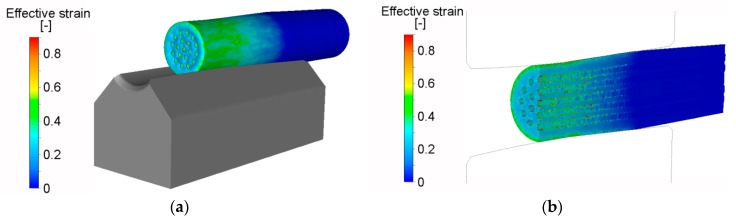
Scalar distribution of effective imposed strain (**a**); effective strain along axial longitudinal cut (**b**).

Numerical analysis of the first swaging reduction of the Al/Cu composite was performed in order to determine possible differences in the deformation behaviours of both metal elements. As seen in Figure 2a, the axial plastic flow of the Al_sheath_ was already significant during the first swaging pass, whereas the plastic flow of the Cu_wires_ was not as substantial (similar behaviour has been documented before, for an Al/Cu clad composite with a different stacking sequence [45]). This phenomenon can primarily be attributed to two factors. The first is related to the total amount and distribution of the imposed strain. As evident from Figure 2a and the axial longitudinal, cut through the composite depicted in Figure 2b, the maximum imposed strain was localized in the (sub) surface region of the composite, i.e., within the Al_sheath_. However, the maximum values of the imposed strain, at approximately 0.6, were also detected at the mutual Al_sheath_/Cu_wires_ interface.

**Figure 3:**
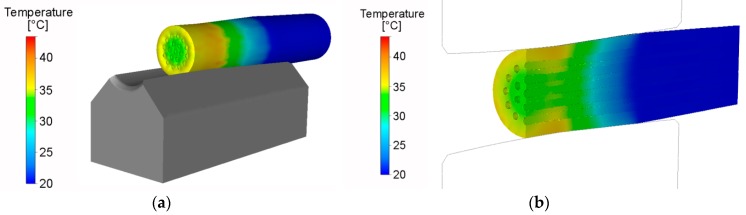
Distribution of temperature throughout *20C* swaged composite: surface (**a**); axial longitudinal cut (**b**).

The second factor affecting plastic flow is related to processing temperature. Despite the fact that RS was performed at room temperature, temperature increased rapidly during swaging, due to the development of deformation heat generated primarily by the movement of atoms, generation of lattice distortions, and nucleation of structural defects [46]. Figure 3a shows that the maximum temperature of the swaged piece increased by 70% on input to the swaging dies’ reduction zone. Similar to imposed strain, the temperature exhibited gradient distribution from the swaged piece surface towards its axis. However, as documented by Figure 3b, the temperature gradient, caused by the effect of deformation heat, was not linear throughout the cross-section of the composite, due to the different physical properties of both the metal elements; the temperature in the axial composite region was higher for the Al_sheath_ than for the Cu_wires_. However, the temperature field exhibited a tendency to homogenize during subsequent swaging.

**Figure 4:**
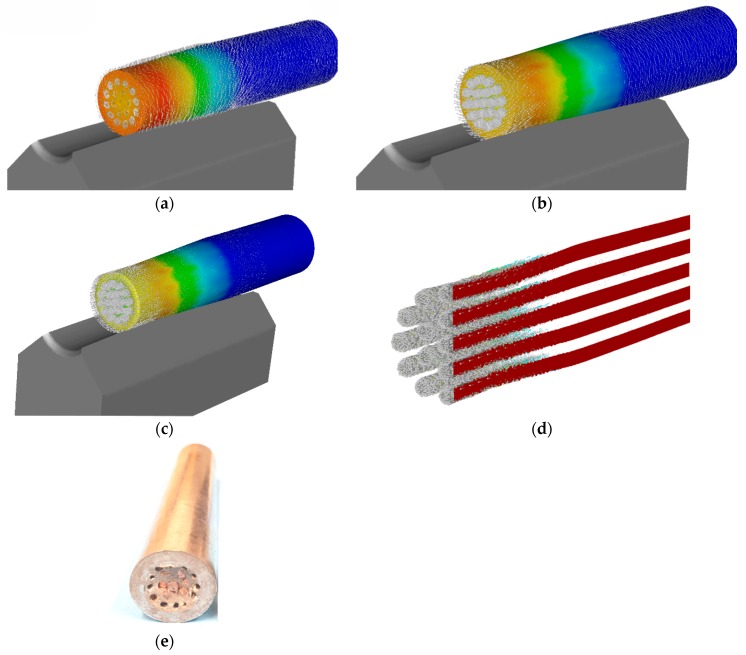
Vector profiles of plastic flow for composite *20C*: dies in contact with swaged-piece (**a**); dies closing and rotating (**b**); dies fully affecting the swaged piece (**c**); plastic flow of Cu_wires_ (**d**); *20C* experimental composite after first swaging pass (**e**).

Due to the effect of the aforementioned phenomena, and the rather complex mutual influence of the axial and tangential swaging force components [8], the plastic flow was not only different for both composite elements, but also changed its character during the swaging pass. At the beginning of the swaging pass, at the moment the dies came into the contact with the material, the material experienced a solely tangential flow (Figure 4a). The tangential plastic flow component then dominated the axial flow component, as the dies came into full contact with the material (Figure 4b). The entire closure of the swaging head then caused the axial plastic flow to dominate the tangential flow component (Figure 4c). The dominant axial plastic flow of the Al_sheath_ suppressed the axial plastic flow of the Cu_wires_, as seen in Figure 4d. This phenomenon indicates that the plastic flow of the Al_sheath_ switched toward the unswaged part of the piece, due to the mutual influence of the varying effects of both the swaging force components and the geometry (conical shape) of the swaging dies reduction zone.

**Figure 5:**
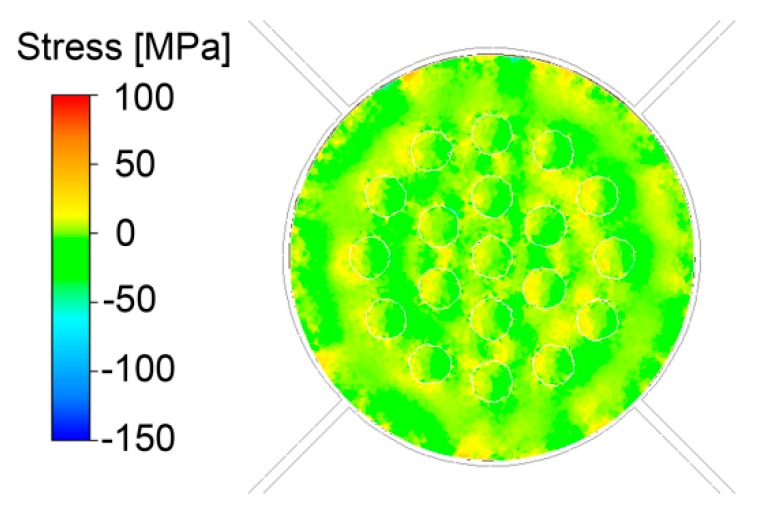
Predicted distribution of internal stress throughout *20C* composite cross-section.

The different deformation behaviours of both metal elements influenced the stress–strain conditions within the swaged piece, as well. Since rotary swaging promotes mutual bonding of the individual interfaces, via mechanical locking and diffusion bonding [47], the incremental character of the imposed strain especially affected the local stress state. Although the cross-sectional cut of the swaged piece exhibited prevailing compressive stress, locations featuring tensile stress were also present. However, their occurrence was primarily limited to the Al_sheath_/Cu_wires_ interfaces and selected regions of the composite surface area (Figure 5). The maximum values of both the stress components ranged within ±50 MPa.

### 3.2. Swaging Parameters

**Figure 6:**
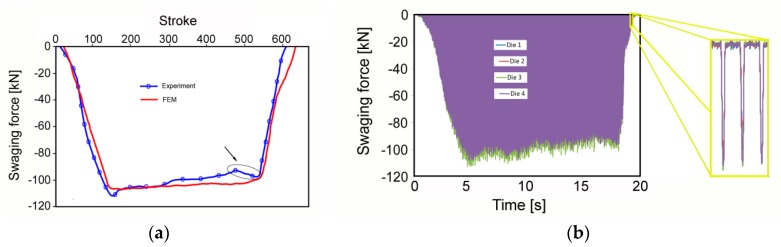
Comparison of numerically predicted and dynamically measured swaging force developments (**a**); dynamically measured swaging forces for *20C* composite (**b**).

The numerically predicted load—swaging force—was subsequently verified via dynamic measurement of the experimental swaging forces. Mutual comparison of the numerically predicted and experimentally measured development of forces during cold swaging exhibits a satisfactory correlation (Figure 6a). However, certain differences between the two curves can be observed. Figure 6a shows an increase in force at approximately 500 strokes (i.e., 500 hits of the swaging dies to the swaged piece), which can be attributed to changes in the plastic flow (most likely related to the increasing influence of the unswaged end [45]), which aggravates the flow of the swaged material out of the swaging dies, and consequently increases the flow stress (mutual comparison of the predicted and real composite plastic flow can be shown using Figure 2 and Figure 4). Nevertheless, most of the differences between the two swaging force developments were related to slight variations in the input parameters—factors such as remeshing, defined thermo-physical properties of the used materials, or tribological conditions.

The measuring system not only allows assessment of the overall load of the machine, but also the individual monitoring of each swaging die; the detail in Figure 6b shows the peak forces for all four swaging dies during several individual strokes. The measured individual forces document homogeneous load, i.e., the homogeneity of the imposed strain, throughout both the composite length and cross-section.

### 3.3. Residual Stress and Grain Size

**Figure 7:**
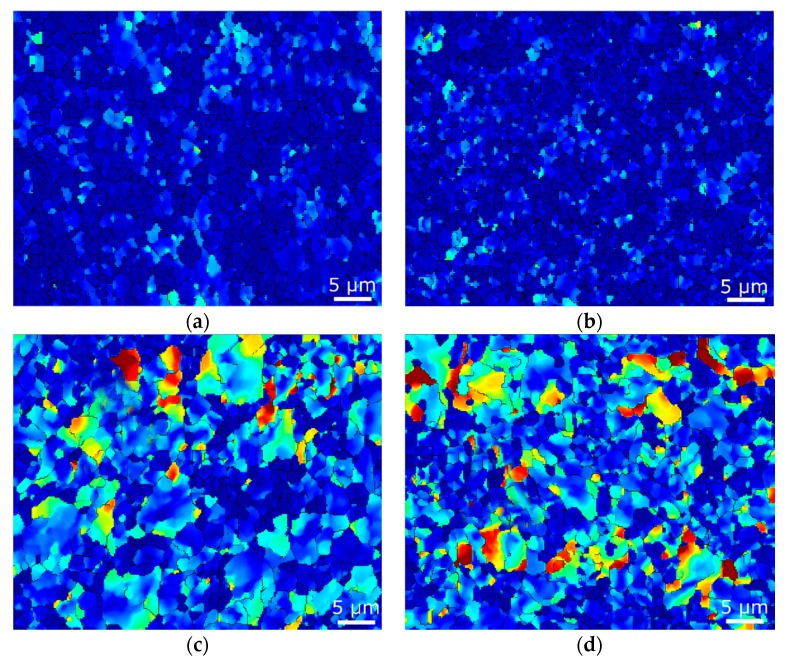
Maps of internal grain misorientation, indicating distribution of residual stress for *20C* composite: Al_sheath_ (**a**); Cu_wire_ (**b**); *250C* composite—Al_sheath_ (**c**); Cu_wire_ (**d**).

Characterization of residual stress within layered multi-metallic composites is especially important, due to the different properties of individual element metals. The analyses were performed via the evaluation of internal grain misorientation on the EBSD scans, acquired in the locations depicted in Figure 1a, on the rainbow scale from blue (0°), to red (15°). The distribution of the internal misorientation points to the distribution of residual stress. As seen in Figure 7a,b, both the Al_sheath_ elements featured restored ultra-fine grains (UFG), with a low amount of misorientation (prevailing blue colour). The average Al_sheath_ grain areas for the *20C* and *250C* composites were 0.62 and 0.39 μm^2^, respectively. On the other hand, internal misorientations were observed within the Cu_wires_ of both composites (Figure 7c,d). The grain size analyses of Cu_wires_ revealed the Cu grains to be larger than the Al grains; the average grain areas were 2.1 μm^2^ for composite *20C*, and 2.9 μm^2^ for composite *250C*. 

### 3.4. Texture

**Figure 8:**
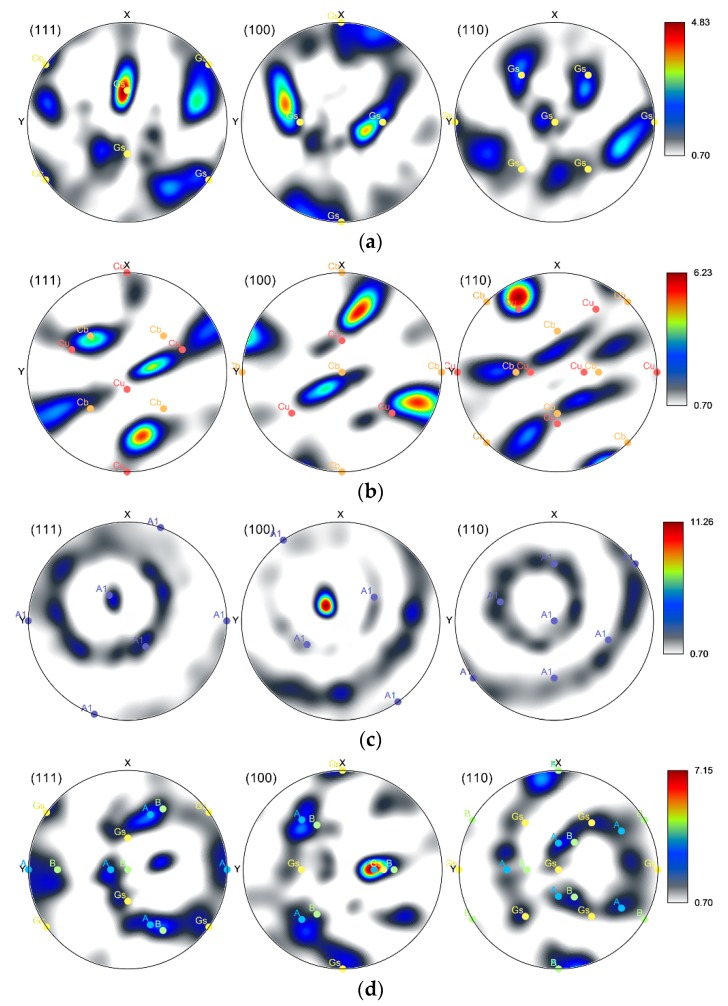
Pole Figures for Al_sheath_ of composite 20C (**a**); composite 250C (**b**); Cu_wires_ of composite 20*C* (**c**); composite 250*C* (**d**).

Figure 8a–d show the (111), (100), and (110) pole figures (PF) for the Al_sheaths_ and Cu_wires_ of both the *20C* and *250C* composites. In the figures, relevant distinctive shear [48], recrystallization and rolling [49] ideal orientations are also depicted. As shown, the textures of Al_sheath_ in both composites feature preferential orientations typical of rolled and recrystallized FCC metals. The Al_sheath_ of the *20C* composite exhibited the tendency to form the ideal *Goss* texture, the characteristic points of which are depicted by *Gs* symbols in the PFs in Figure 8a. As shown in Figure 8b, the Al_sheath_ of the *250C* composite also exhibited the tendency to form ideal texture fibres. The highest intensities were found for the *Copper* (*Cu*) and *Cube* (*Cb*) orientations, however, ideal orientations were shifted by 10°. The Cu_wires_ of both the *20C* and *250C* composites featured a tendency to form ideal shear texture components. The *20C* composite Cu_wires_ featured the *A1* ideal shear texture fibre (Figure 8c), however, the (100) PF also exhibited a strong peak not corresponding to any ideal orientation; it was shifted by about 10° from the ideal *A* fibre shear texture, and by about 15° from the *Cube* and *Copper* ideal orientations. Lastly, Figure 8d documents the effects of swaging on the *250C* composite Cu_wires_. The grains exhibited a tendency to form both the rolling/recrystallization (*Gs*) and shear (*A*, *B*) ideal texture orientations.

## 4. Discussion

The differences in the plastic flows of both the metal elements can non-negligibly influence the overall performance of the final swaged composite. As previously reported, material flow during swaging is primarily influenced by the two main swaging force components, tangential and axial [8]. At the beginning of a swaging pass, the tangential plastic flow component dominates the axial one, but the axial flow component starts to dominate the tangential one during continuing swaging [45]. However, none of these components are equal to zero during the swaging pass, as documented by the predicted and dynamically measured swaging force developments. Figure 6a,b show that swaging force exhibited a rapid increase, followed by a more or less steady period, and a subsequent slight decrease. The rapid increase in force corresponded to the gradual filling of the swaging head with the unswaged material, and the decrease in the peak force was caused by two main influencing factors, the first of which was a decrease in composite flow stress, due to the influence of increased temperature (caused by the development of deformation heat), and the second related to the incremental character of the swaging process, and the increasing influence of the unswaged end [45]. 

According to the results of numerical prediction, the axial plastic flow of the Al_sheath_ was dominant and suppressed the axial plastic flow of the Cu_wires_. This was one of the main reasons for the occurrence of high effective strains in the (sub)surface composite regions and at the mutual interfaces. The latter was primarily caused by the effect of shear strain, which developed as a result of the mutual sliding of the “softer” Al_sheath_ and the “harder” Cu_wires_. In other words, the substantial axial plastic flow of the Al_sheath_ was imparted by the compressive effect of the swaging dies, which was most significant in the (sub)surface region, as this was in direct contact with the dies. The increased imposed strain at the Al_sheath_/Cu_wires_ interfaces resulted from differences in the material flows of both component metals. On the other hand, the differences in the Al_sheath_/Cu_wires_ interfaces diminished during continuing swaging, due to the increased quality of the mutual bonding of the elements, and the effect of the deformation hardening of both. 

The differences in the plastic flows of the composite elements imparted differences in their structural orientation. As shown by the texture analyses, the grains within the Al_sheaths_ of both the *20C* and *250C* composites exhibited a tendency to form ideal rolling texture orientations, which corresponds to the predicted dominant axial plastic flow. However, the Al_sheath_ of the *250C* composite also featured the *Cube* texture orientation, which is typically found in recrystallized aluminum [49]. This finding corresponds to the results of grain analyses, which revealed that the *250C* composite Al_sheath_ featured the finest grains, with no substantial misorientations indicating structural relaxation during recrystallization, resulting in the diminished presence of residual stress. On the other hand, the grains within both the Cu_wires_ elements exhibited a tendency to form ideal shear texture orientations, which corresponds to the aggravated axial plastic flow and the dominant effect of the tangential swaging force component. Mutual comparison of the textures of the *20C* and *250C* composites shows that swaging temperature affected grain orientation, primarily by imparting changes in the flow stress of both elements, and by supporting restoration. While the dominant Al_sheath_ ideal texture components changed from *Goss* (*20C*) to *Cube* and *Copper* (*250C*), the Cu_wires_ texture components changed from the dominant *A1* fibre (*20C*) to a combination of *A* and *B* fibres and *Goss* ideal orientation (*250C*). The composite metal elements also exhibited strong intensity of orientation, which shifted by 10°–15° from the ideal texture orientations. This can be attributed to the effect of the rotating swaging dies, and the influence of the transversal swaging force component.

The effect of the individual swaging force components manifested not only as grain orientation changes, but also as grain refinement, and changes in the occurrence of residual stress. As the swaging dies affected the swaged piece with compressive stresses throughout the process, the character of the predicted stress distribution throughout the composite cross-section was more or less symmetrical (Figure 4). Nevertheless, differences can be observed in the microscale. As shown in the grain analyses, the dominant plastic flow of the Al_sheath_ imparted a more intense grain refinement within this element. Although both the Al_sheaths_ featured UFG structures with residual stress-free recrystallized grains, the higher swaging temperature resulted in more effective grain refinement. Misorientations were observed within both the *20C* and *250C* composites Cu_wires_ elements—green, yellow, and red areas within the Cu grains point to the development of dislocation tangles and cells, dislocation walls and subgrains, and to the presence of low angle grain boundaries (LAGBs) [50]. The differences in the presence of residual stress within the Al_sheath_ and Cu_wires_ composite elements can be attributed not only to differences in their plastic flows, but also to the intrinsic properties of both metals. Al features high stacking fault energy (SFE), which enables the grains to be easily deformed via dislocation slip, and facilitates the development of restoration processes, and consequent grain refinement and relaxation of internal stresses [49]. On the other hand, the medium SFE of copper imparts shear band formation, results in the development of shear texture, and aggravates the ability of the grains to relax internal stresses. Mutual comparison of the occurrences of residual stress in the Al_sheath_ and Cu_wires_ of the *20C* and *250C* composites did not reveal any significant effects of swaging temperature on this factor. The grains within the Al_sheath_ had already exhibited restoration during swaging at 20 °C, and the increase in temperature to 250 °C only resulted in further grain refinement. For the Cu_wires_, 250 °C was likely insufficient to substantially affect the restoration processes; it only resulted in slight grain growth (i.e., secondary recrystallization [46]).

## 5. Conclusions

This research study dealt with the numerical and experimental evaluation of the presented 5 mm clad composites, consisting of Al_sheath_ and Cu_wires_ fabricated via rotary swaging at 20 °C and 250 °C. The numerical predictions showed the differences in the plastic flow of both the Al_sheath_ and Cu_wires_ to have a major influence on residual stress and grain character, as well as the distribution of the imposed strain. The highest effective strain was observed in the composite (sub) surface region and at the mutual interfaces of the elements, both primarily due to the direct impact of shear strain. Axial plastic flow was dominant for the Al_sheath_, which imparted ideal rolling texture orientations, as well as restoration and grain refinement. The Al_sheath,_ deformed at 250 °C, exhibited especially fine (average area of 0.39 μm^2^) stress-free grains. On the other hand, the axial plastic flow of the Cu_wires,_ featuring higher flow stress, was aggravated; the grains within this element exhibited evidence of residual stress and typical ideal shear texture orientation. Although the increased temperature supported axial flow (changes in texture orientation), it was not sufficient to introduce restoration, only resulting in slight secondary grain growth—the average grain areas within the Cu_wires_ were 2.1 μm^2^ for composite *20C*, and 2.9 μm^2^ for composite *250C*.

## Figures and Tables

**Table 1:** Swaging reductions in individual passes for both 20*C* and 250*C* composites.

**Pass No.**	1	2	3	4	5	6
**Swaging Degree *φ***	0.36	0.81	1.38	2.20	2.77	3.58

**Table 2:** Thermo-physical parameters for investigated material used for simulation.

Property	Unit	Al	Cu
Poisson Coefficient	-	0.3	0.3
Density	(g.cm^−3^)	2.7	8.9
Specific Heat	(J.kg^−1^.K^−1^)	1230	398
Thermal Conductivity	(W/(m.K))	204	386
